# Factors Associated With Health Literacy Among Women in Menopause in Western Iran: A Cross-Sectional Study

**DOI:** 10.3928/24748307-20260622-01

**Published:** 2026-10

**Authors:** Ensiyeh Jenabi, Salman Khazaei, Sara Abdoli, Sanaz Javadiyeganeh

**Affiliations:** a Mother and Child Care Research Center, Institute of Health Sciences and Technologies, Avicenna Health Research Institute; b Department of Epidemiology, Research Center for Health Sciences and; c Student Research Committee, Hamadan University of Medical Sciences, Hamadan, Iran.

## Abstract

**Background:**

There is a lack of information regarding health literacy in homemaker menopausal women in the western Iran. The aim of this study was to investigate factors associated with health literacy among women in menopause who are homemakers in western Iran.

**Methods:**

This cross-sectional study was conducted from February 5, 2025 to August 20, 2025, among 350 women in menopause attending comprehensive health service centers in Hamadan, western Iran. The participants were recruited via systematic random sampling. The data collection instruments consisted of a demographic and midwifery information questionnaire, the Health Literacy for Iranian Adults, the Perceived Stress Scale, and the Multidimensional Scale of Perceived Social Support. A *p* value *of* < .05 was considered statistically significant. Independent *t*-tests and one-way ANOVA (analysis of variance) were used to compare mean health literacy across categorical variables. Pearson's correlation coefficient was applied to examine the relationships among social support, perceived stress, and health literacy. In addition, to identify independent predictors of health literacy, a multivariable linear regression model was constructed. All analyses were conducted using Stata version 14.

**Results:**

The health literacy levels show that the majority (267; 76.29%) have inadequate health literacy. The mean health literacy was significantly associated with participants' education (*p* < .001), occupation (*p* < .001), husbands' occupation (*p* < .001), and economic status (*p* < .001). The significant negative correlation was observed between health literacy and perceived stress (*r* = −0.86), suggesting that higher health literacy was associated with lower levels of perceived stress. Moreover, health literacy was positively correlated with social support (*r* = 0.77), indicating that individuals with greater health literacy tended to report higher levels of social support. The multivariable linear regression model identified higher perceived stress (β = −2.05, *p* < .001) and older age (β = −1.40, *p* = .03) as significant negative predictors of health literacy, while greater social support (*β* = 1.46, *p* < .001) was a significant positive predictor.

**Conclusion:**

The findings of the present study revealed that the majority of the women had an inadequate level of health literacy, and this variable had a negative relationship with perceived stress and a positive relationship with social support. The results of this research emphasize the importance of psychosocial factors in promoting health literacy women in menopause. Perceived stress and social support are two key variables that can either facilitate or hinder the path to understanding and utilizing health information. Designing interventions aimed at reducing stress and strengthening social support, and examining their impact on health literacy, could enrich the empirical knowledge base. Furthermore, investigating the role of mediating variables such as health self-efficacy, self-esteem, coping styles, and attitudes toward health could lead to a deeper understanding of the psychological mechanisms affecting health literacy. Qualitative or mixed-methods research would also be capable of clarifying the cultural dimensions, lived experiences, and subjective meanings associated with menopause, providing a foundation for designing localized educational programs.

## Abstract (plain language summary)

**Plain Language Summary: **Most women in menopause in this study had inadequate health literacy. Better health literacy was linked to higher education, employment, and better economic status. Women with higher health literacy reported less stress and more social support. Higher stress and older age predicted lower health literacy, while greater social support predicted better health literacy.

Menopause is a natural biological process that marks the end of a woman's reproductive years, typically occurring between ages 45 and 55 years (Gatenby & Simpson, 2024). Although menopause is a physiological phenomenon, it is often accompanied by numerous physical, psychological, and social changes that can significantly affect a woman's quality of life (Hickey et al., 2022). These changes—including hot flashes, sleep disturbances, mood swings, and reduced concentration—require an adequate level of health knowledge and adaptive behaviors to adjust to the new conditions (Davis et al., 2023). Therefore, health literacy has been introduced as one of the key factors determining health outcomes in women in menopause (Lee & Kim, 2024).

Health literacy refers to an individual's ability to obtain, process, and understand basic health information and services needed to make appropriate health decisions (Nutbeam & Lloyd, 2021). Health literacy helps individuals to effectively navigate the health care system, participate in preventive activities, and choose healthier lifestyles (Jung et al., 2022).

Among women in menopause, nadequate health literacy may lead to poor management of menopausal symptoms, inappropriate use of hormonal or herbal treatments, and adverse mental health outcomes (Khandehroo et al., 2022). Studies have shown that self-care education tailored with a health literacy index has a significant impact on increasing self-care behaviors and improving the quality of life for menopausal women; a study by Hossein Mirzaee Beni et al. (2022) found that education based on a health literacy index led to a significant increase in self-care behaviors and the quality of life for women in menopause. Malekmirzaei et al. (2023) also reported that women in who are postmenopausal with higher health literacy engage in better preventive self-care behaviors and have a lower risk of developing osteoporosis. Recent research findings indicate that health literacy is influenced by a combination of demographic, psychological, and social factors. Variables such as age, education level, economic status, and access to health care services have been reported as factors associated with health literacy (Abdolmalaki et al., 2018).

However, beyond these factors, psychosocial variables such as social support and perceived stress also play a significant role in shaping and promoting health literacy. Social support and perceived stress are among the psychosocial factors that may determine the level of health literacy. A study in India showed that social support can reduce the severity of menopausal symptoms, and women with stronger support experience better levels of mental and physical health (Divya et al., 2024). Furthermore, Arnot et al. (2021) examined the relationship between stressful events, social support, and menopausal symptoms and found that when women experienced a stressful event, the severity of hot flashes was reported to be higher.

In many developing countries, older adult women face numerous cultural and structural barriers in accessing health information and services. Cultural taboos surrounding menopause, low levels of general literacy, and gender inequalities in health education can exacerbate these challenges (Takenaka & Sakai, 2021). Therefore, investigating the predictors of health literacy during menopause is not only an individual matter but also a social issue and a step toward health equity and gender-sensitive, health-focused care.

Despite increasing global attention to the importance of health literacy, few studies have investigated its determining factors in women during menopause. Most existing research has focused on the general adult population or patients with chronic diseases, leaving a significant research gap concerning women in their postreproductive years. Furthermore, only a limited number of studies have simultaneously examined the roles of social support and perceived stress as factors associated with health literacy in this specific group.

Investigating these relationships concurrently can provide valuable insights for health professionals and policy-makers in developing strategies to promote women's health. Given the cultural context of western Iran, no study has yet examined the factors associated with health literacy among menopausal homemakers. Therefore, the aim of this study was to investigate factors associated with health literacy among women in menopause who are homemakers in western Iran. The findings of this study could be effective in improving health literacy, promoting self-care behaviors, and enhancing the overall well-being of women in menopause.

## Materials and Methods

### Setting and Design of the Study

This cross-sectional study was conducted from February 5, 2025 to August 20, 2025, among 350 women in menopause who are homemakers attending comprehensive health service centers in Hamadan, western Iran. Hamadan was divided into four geographical regions, and four centers were randomly selected from each region. Participants were then recruited through systematic random sampling. After explaining the study objectives, researchers obtained written informed consent from all participants. The study protocol was approved by the Ethics Committee of Hamadan University of Medical Sciences with Code IR.UMSHA. REC.1404.027.

### Inclusion and Exclusion Criteria

The inclusion criteria were women age 45 to 60 years, with natural menopause, and between 1 and 5 years since their last menstrual period. Exclusion criteria included a history of hormone therapy, hysterectomy, or oophorectomy, as well as the presence of chronic physical or mental illnesses.

### Sample Size

Based on the findings of Abdolmalaki et al. (2018), in which 35% of participants demonstrated a desirable level of health literacy, and considering a type I error rate of 0.05 with a test precision of 0.05, the required sample size was calculated as 350 women.

### Measurement Tools

The data collection instruments consisted of a demographic and midwifery information questionnaire, the Health Literacy for Iranian Adults (HELIA), the Perceived Stress Scale (PSS), and the Multidimensional Scale of Perceived Social Support (MSPSS). All questionnaires were administered through researcher-conducted interviews.

***Demographic and Midwifery Information Questionnaire.*** This questionnaire collected information on age, age at menopause, duration of menopause, education, occupation, husband's occupation, number of children, number of abortions, number of stillbirths, economic status, housing situation, and insurance status.

***HELIA.*** The development and psychometric properties of this questionnaire were established by Montazeri et al. (2014), reporting Cronbach's alpha coefficients ranging from 0.72 to 0.89. The questionnaire comprises two main parts. The first part addresses respondent characteristics, including age, gender, marital status, literacy level, and sources of health information. The second part consists of 33 items across five subscales: access to health information (6 items), reading ability (4 items), comprehension (7 items), evaluation (4 items), and decision-making and application of information (12 items). All items are rated on a five-point Likert scale (1 = *strongly disagree* to 5 = *strongly agree*). The possible health literacy score ranges from 33 to 165, with levels classified into four categories: inadequate (0 to 50), marginal (51 to 66), adequate (67 to 84), and excellent (85 to 100).

***PSS.*** The PSS (PSS-14) consists of 14 items rated on a 5-point Likert scale ranging from *never* to *very often*, and evaluates perceived stress experienced during the past month (Cohen et al., 1983). Example items include: “In the past month, how often have you felt unable to control the important things in your life?”, “In the past month, how often have you felt confident about your ability to handle personal problems?”, and “In the past month, how often have you felt that things were going your way?” The total score ranges from 0 to 56, with higher scores reflecting greater levels of perceived stress. The psychometric properties of the PSS-14 were validated in Iran by Sharifi-Nia et al. (2024), who reported a reliability coefficient exceeding 0.70.

***MSPSS.*** MSPSS consists of 12 items rated on a 5-point Likert scale ranging from 1 (*strongly disagree*) to 5 (*strongly agree*). The items assess perceived social support from three sources: family, friends, and significant others. Example items include: “My family really tries to help me” and “I get the emotional help and support I need from my family.” Higher scores reflect greater perceived social support. The total score is classified into three levels: low (1.00 to 2.33), moderate (2.34 to 3.67), and high (3.68 to 5.00) (Zimet et al., 1988). The psychometric properties of the Persian version of the questionnaire were previously evaluated by Bagharian et al. (2013), reporting a reliability of 0.84. In the present study, the scale demonstrated excellent internal consistency, with a Cronbach's alpha of 0.91.

### Statistical Analyses

The normality of quantitative variables was assessed using the Kolmogorov-Smirnov test. For variables with a normal distribution (*p* > .05), descriptive statistics were presented as mean ± standard deviation. Independent *t*-tests and one-way ANOVA were used to compare mean health literacy across categorical variables. Pearson's correlation coefficient was applied to examine the relationships among social support, perceived stress, and health literacy. To identify independent predictors of health literacy, a multivariable linear regression model was constructed, including variables that showed significant associations in the univariate analyses. The variance inflation factor (VIF) was calculated to check for multicollinearity among the predictor variables in the regression model, with a V*IF* < 5 considered acceptable. A *p* value *of* < .05 was considered statistically significant. All analyses were conducted using Stata version 14.

## Results

A total of 387 women were invited to participate, yielding a final sample of 350 respondents and a response rate of approximately 90.4%. Their mean (± standard deviation [SD]) age was 52.06 ± 3.44 years, the mean age at menopause was 48.59 ± 3.03 years, and the mean duration of menopause was 3.49 ± 1.33 years. The mean number of children was 3.69 ± 1.14, while the mean (±SD) numbers of abortions and stillbirths were 0.96 ± 0.89 and 0.46 ± 0.65, respectively (**Table 1**). The health literacy levels among them show that the majority (267; 76.29%) have inadequate health literacy, with smaller proportions categorized as relatively adequate (41; 11.71%), adequate (36; 10.29%), and excellent (6; 1.71%) (**Table 2**).

**Table 1:** Demographic (Qualitative) Characteristics Among Women in Menopause

**Variable**	**Categorize**	***n***	**%**
Education	Primary	76	21.71
	High school	149	42.57
	Diploma	89	25.43
	University	36	10.29
Occupation	Homemaker	329	94.00
	Employee	21	6.00
Husband's occupation	Unemployed	39	11.14
	Self-employed	165	47.14
	Employee	35	10.00
	Manual worker	111	31.71
Economic status	Low income (<15 Million Rials [<$180] per month)	100	28.57
	Moderate income (15–30 Million Rials [$180–$360] in month)	250	71.43
Insurance status	With insurance	307	87.71
	Without insurance	43	12.29
Housing situation	Private	76	21.71
	Mortgage and rent	200	57.14
	Living with relatives	74	21.14

**Table 2:** Correlation of Mean Health Literacy With Demographic Characteristics Among Women in Menopause

**Variable**		**Mean ± *SD***	***F***	***p***
Education	Primary	59.09 ± 21.43		
	High school	70.26 ± 30.13	17.81	<.001
	Diploma	68.55 ± 29.07		
	University	99.89 ± 26.47		
Occupation	Homemaker	68.12 ± 28.98	36.89	<.001
	Employee	106.86 ± 14.11		
Husband's occupation	Unemployed	61.41 ± 26.18		
	Self-employed	72.53 ± 30.51	6.73	<.001
	Employee	87.40 ± 28.88		
	Manual worker	65.18 ± 27.78		
Economic status	Low income	57.55 ± 19.74	28.37	<.001
	Moderate income	75.61 ± 31.50		
Insurance status	With insurance	72.69 ± 30.89	14.70	<.001
	Without insurance	54.47 ± 10.45		
Housing situation	Private	102.41 ± 30.19	89.94	<.001

Relationships between stress, social support, and health literacy and demographic characteristics among women are presented in **Table 3**. Stress was significantly correlated with age (*r* = 0.44), husband's age (*r* = 0.31), age at menopause (*r* = 0.24), duration of menopause (*r* = 0.24), number of children (*r* = −0.13), number of abortions (*r* = −0.14), and number of stillbirths (*r* = −0.15). Social support was significantly correlated with age (*r* = −0.41), husband's age (*r* = −0.31), age at menopause (*r* = −0.22), duration of menopause (*r* = −0.45), number of children (*r* = 0.11), and number of abortions (*r* = 0.15). Health literacy was significantly correlated with age (*r* = −0.85), husband's age (*r* = −0.49), age at menopause (*r* = −0.25), duration of menopause (*r* = −0.54), number of abortions (*r* = 0.17), and number of stillbirths (*r* = 0.17). No significant associations were observed between social support and number of stillbirths (*p* = .10) or between health literacy and number of children (*p* = .10) (**Table 3**).

**Table 3:** Bivariate Correlations of Stress, Social Support, and Health Literacy Among Women in Menopause

**Variables**	**Age**	**Husband's Age**	**Menopause Age**	**Menopause Duration**	**Number of Children**	**Number of Abortions**	**Number of Stillbirths**	**Perceived Stress**	**Social Support**	**Health Literacy**
Age	1	0.49**	0.85**	0.50**	−0.11*	−0.10*	0.00	0.44**	−0.41**	−0.47**
Husband's age		1	0.42**	0.33**	−0.07	−0.16**	0.06	0.31**	−0.31**	−0.29**
Menopause age			1	0.12*	−0.07	−0.05	0.03	0.24**	−0.22**	−0.25**
Menopause duration				1	−0.09	−0.11*	−0.05	0.52**	−0.45**	−0.54**
Number of children					1	0.09	−0.01	−0.13*	0.11*	0.10
Number of abortions						1	0.02	−0.14*	0.15**	0.17**
Number of stillbirths							1	−0.15*	0.10	0.17**
Perceived stress								1	−0.74**	−0.86**
Social support									1	0.77**
Health literacy										1

* p < .05;

** p < .001

**Table 4** presents the results of a multivariable linear regression analysis identifying predictors of health literacy. The model shows that higher perceived stress was significantly associated with lower health literacy scores (β = −2.05, *p* < .001), while greater social support was significantly associated with higher scores (*β* = 1.46, *p* < .001). Older age was also a significant negative predictor (β = −1.40, *p* = .03). Other variables, including spouse's age, age at menarche, duration of menopause, and number of children, were not statistically significant predictors in this model.

**Table 4:** Multivariable Linear Regression Analysis for Predictors of Health Literacy

**Predictor Variable**	**Coefficient (β)**	**Standard Error**	***p***	**95% CI**
Age	−1.40	0.64	.03	[−2.67, −0.14]
Spouse's age	0.34	0.22	.12	[−0.09, 0.77]
Age at menarche	0.82	0.63	.20	[−0.42, 2.06]
Duration of menopause	−1.29	0.89	.15	[−3.0, 0.47]
Number of children	1.27	0.83	.13	[−0.36, 2.89]
Perceived stress	−2.05	0.13	<.001	[−2.31, −1.79]
Social support	1.46	0.20	<.001	[1.06, 1.86]

Note. CI = confidence interval.

## Discussion

The findings of the present study revealed that the majority of the women had an inadequate level of health literacy, and this variable had a negative relationship with perceived stress and a positive relationship with social support. This pattern indicates that health literacy in women during menopause is not merely an individual cognitive skill but a multidimensional phenomenon influenced by psychological and social contexts. Accordingly, the findings suggest that improving health literacy in these women requires addressing the psychosocial level, and relying solely on cognitive or informational education is insufficient.

The present study aligns with the findings of Abdolmalaki et al. (2018) in Iran, confirming that a significant proportion of women in menopause have inadequate health literacy. At the same time, the inconsistency with the results of Jenabi et al. (2020) in Iran, who reported a relatively more favorable level of health literacy, likely reflects environmental and cultural differences (Jenabi et al., 2020). Specifically, in urban areas with greater access to media and health services, public awareness may be higher (Chen et al., 2019).

These differences highlight the necessity of analyzing health literacy within specific cultural and economic contexts (Shaw et al., 2009). This is because the meaning and application of health knowledge vary across cultures and are dependent on social beliefs about illness, health, and gender roles (Bindhu et al., 2024).

The positive correlation between education level and health literacy is consistent with the findings of Nezafati et al. (2020), who identified low education as the most significant predictor of inadequate health literacy. From a cognitive psychology perspective, higher education is typically associated with stronger analytical skills, critical thinking, and a better understanding of scientific concepts, all of which are effective in comprehending health messages (Dwyer, 2023).

Furthermore, women with higher education usually utilize more diverse resources to access health information and rely less on traditional beliefs or nonscientific sources (Dong et al., 2023). These findings indicate that promoting women's education and improving the general literacy of society can indirectly lead to improved health literacy and a reduction in health disparities.

On the other hand, the observed association between occupation and health literacy in this study can be explained through two pathways. First, from an economic perspective, stable employment is usually associated with higher income and, consequently, greater access to health services and resources (Svendsen et al., 2021). Second, from a sociocognitive perspective, the work environment increases opportunities for social interaction, exchange of experiences, and informal learning (Ehmann et al., 2021).

The findings of Mosallanezhad et al. (2019), which highlighted the role of the “cognitive burden of poverty” in diminishing mental capacity for health decision-making, support this conclusion. Chronic poverty limits not only economic resources but also an individual's psychological and cognitive resources, and the stress it induces impairs the ability to concentrate and process information (Blair & Raver, 2016).

Regarding the variable of stress, the results of the present study are consistent with the findings of Michou et al. (2021), which showed that low health literacy is associated with high perceived stress. There are some studies in Iran, in the other group of participants in the field of health literacy and women, health behavior and social support. A cross-sectional study was conducted performed 2023 in Iran on 259 among married women. This study investigated cognitive factors and health literacy as determinants of Pap smear screening. The differences in Pap smear screening by perceived barriers as cognitive factor were statistically significant. The women who perceived fewer barriers and higher level of self-efficacy to conduct the test were more likely to have a Pap smear screening (Babazadeh et al., 2025). A cross sectional study examined 379 adult residents of the city and evaluated the relationship between health literacy and health promotion behaviors (Ranjbaran et al., 2022). The average health literacy level was moderate. A positive and significant association between health literacy and health promotion behaviors was observed. The strongest determinants of higher health literacy were higher education and higher income. Educational programs, especially for groups with low literacy, should focus on strengthening decision-making skills and improving access to health resources (Ranjbaran et al., 2022). In another study, receiving social support among cardiovascular patients was directly positively associated with quality of life; patients with higher levels of social support reported better quality of life and experience reduced feelings of depression and anxiety. Positive social support predicted quality of life in patients with heart disease. The effect of this support was mediated by reductions in symptoms of depression and anxiety (Chollou et al., 2022). It can be hypothesized that low health literacy causes an individual to feel more helpless and anxious when confronted with physical symptoms or conflicting health information.

On the other hand, chronic stress impairs cognitive function, reduces concentration, and diminishes the motivation to learn (Girotti et al., 2024). This bidirectional cycle can gradually lead to a decrease in self-care behaviors and an increased risk of physical and psychological problems. Therefore, reducing stress can improve health literacy not only directly but also indirectly by enhancing cognitive capacity and the motivation to learn (Dehghani, 2024).

The finding regarding the positive association between social support and health literacy is also of significant importance. Social support can be effective in two ways: directly, by providing information, guidance, and healthy behavioral models; and indirectly, by reducing stress and enhancing the sense of self-efficacy (He et al., 2024).

Divya et al. (2024) have demonstrated that positive social interactions strengthen feelings of belonging and psychological security, which ultimately leads to improved health understanding and more active participation in treatment decisions (Divya et al., 2024). In this context, it can be said that health literacy is also a collective skill; because in a supportive environment, individuals not only receive information but also experience deeper learning through observation, exchange of experiences, and social feedback (Jung et al., 2022).

From a policy perspective, the findings of the present study indicate that improving health literacy among women during menopause should be pursued through multilevel, community-based interventions. Educational programs that are purely individual-focused, without considering the psychosocial context, may have limited effectiveness.

Designing group workshops centered on teaching communication skills, stress management, and family interaction can strengthen social support and, consequently, increase health literacy. Furthermore, utilizing digital media and social networks to deliver understandable and culturally-adapted health messages can be an effective tool for empowering middle-aged and menopausal women.

## Study Limitations

One of the study's limitations is its cross-sectional nature, which restricts the ability to infer causality. Self-reported data may also have been influenced by memory bias or the tendency to provide socially desirable responses. Furthermore, confining the study population to a specific geographical and cultural area reduces the generalizability of the results. In addition, lack of measurement of potentially important confounding variables (e.g., self-efficacy, coping styles) was another limitation of this study.

## Conclusion

The findings of the present study revealed that the majority of women in menopause who are homemakers had an inadequate level of health literacy, and this variable had a negative relationship with perceived stress and a positive relationship with social support. The results of this research emphasize the importance of psychosocial factors in promoting health literacy among women. It is recommended that future research employ longitudinal or interventional designs to investigate the causal direction of the relationships. Designing interventions aimed at reducing stress and strengthening social support, and examining their impact on health literacy, could enrich the empirical knowledge base. Furthermore, investigating the role of mediating variables such as health self-efficacy, self-esteem, coping styles, and attitudes toward health could lead to a deeper understanding of the psychological mechanisms affecting health literacy. Qualitative or mixed-methods research would also be capable of clarifying the cultural dimensions, lived experiences, and subjective meanings associated with menopausal, providing a foundation for designing localized educational programs. In addition, future research should further explore diverse populations and settings to enhance the generalizability of the findings and examine other contextual and structural determinants of health literacy. Employing more robust methodological approaches and larger sample sizes could strengthen the evidence base.
